# Peripheral neuropathy in metachromatic leukodystrophy: current status and future perspective

**DOI:** 10.1186/s13023-019-1220-4

**Published:** 2019-11-04

**Authors:** Shanice Beerepoot, Stefan Nierkens, Jaap Jan Boelens, Caroline Lindemans, Marianna Bugiani, Nicole I. Wolf

**Affiliations:** 1 Department of Child Neurology, Emma Children’s Hospital, Amsterdam UMC, Vrije Universiteit Amsterdam, and Amsterdam Neuroscience, De Boelelaan 1117, Amsterdam, the Netherlands; 2 Center for Translational Immunology, University Medical Center Utrecht, Utrecht, the Netherlands; 3 Department of Pediatrics, Stem Cell Transplant and Cellular Therapies, Memorial Sloan Kettering Cancer Center, New York, NY USA; 4 Pediatric Blood and Marrow Transplantation Program, Princess Máxima Center and University Medical Center Utrecht, Utrecht, the Netherlands; 5 Regenerative medicine institute, University Medical Center Utrecht, Utrecht, the Netherlands; 6 Department of Pathology, Amsterdam UMC, Vrije Universiteit Amsterdam, Amsterdam Neuroscience, De Boelelaan 1117, Amsterdam, the Netherlands

**Keywords:** Metachromatic leukodystrophy, *ARSA* gene mutation, Lysosomal storage disorder, Neuropathy, Leukodystrophy, Demyelinating

## Abstract

Metachromatic leukodystrophy (MLD) is an autosomal recessively inherited metabolic disease characterized by deficient activity of the lysosomal enzyme arylsulfatase A. Its deficiency results in accumulation of sulfatides in neural and visceral tissues, and causes demyelination of the central and peripheral nervous system. This leads to a broad range of neurological symptoms and eventually premature death. In asymptomatic patients with juvenile and adult MLD, treatment with allogeneic hematopoietic stem cell transplantation (HCT) provides a symptomatic and survival benefit. However, this treatment mainly impacts brain white matter, whereas the peripheral neuropathy shows no or only limited response. Data about the impact of peripheral neuropathy in MLD patients are currently lacking, although in our experience peripheral neuropathy causes significant morbidity due to neuropathic pain, foot deformities and neurogenic bladder disturbances. Besides, the reasons for residual and often progressive peripheral neuropathy after HCT are not fully understood. Preliminary studies suggest that peripheral neuropathy might respond better to gene therapy due to higher enzyme levels achieved than with HCT. However, histopathological and clinical findings also suggest a role of neuroinflammation in the pathology of peripheral neuropathy in MLD. In this literature review, we discuss clinical aspects, pathological findings, distribution of mutations, and treatment approaches in MLD with particular emphasis on peripheral neuropathy. We believe that future therapies need more emphasis on the management of peripheral neuropathy, and additional research is needed to optimize care strategies.

## Background

**Fig. 1:**
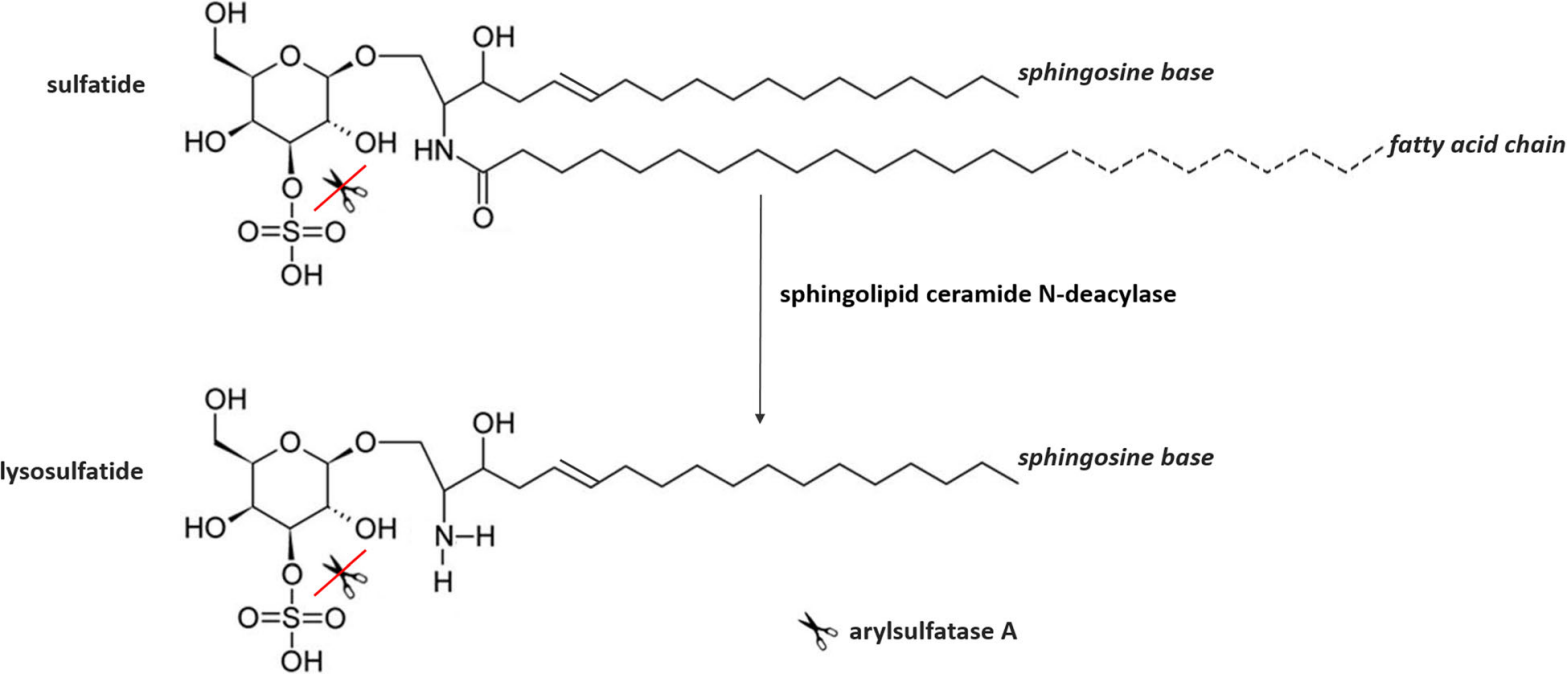
Sulfatide and lysosulfatide structures. Sulfatide (*3-O-sulfogalactosylceramide*) consists of a ceramide backbone (i.e. a long-chain base and a fatty acid chain) and a sulfated galactose moiety. Lysosulfatide (*1-(3-O-sulfo-beta-D-galactosyl)sphingosine*) is the deacylated form of sulfatide [2]. In MLD patients the lysosomal enzyme arylsulfatase A (ASA), which hydrolyzes the sulfate group in the degradation of sulfatide and lysosulfatide, is deficient, leading to accumulation of (lyso) sulfatides in various tissues (e.g. nervous tissue, gall bladder, kidneys and liver) [2]

Metachromatic leukodystrophy (MLD, MIM 250100) is an autosomal recessively inherited metabolic disease caused by deficient activity of the lysosomal enzyme arylsulfatase A (ASA) [1]. This enzyme catalyzes the first step in the degradation of various sulfatides in lysosomes, including 3-*O*-sulfogalactosylceramide (sulfatide) and 1-(3-*O-*sulfo-beta-D-galactosyl) sphingosine (lysosulfatide) [2] (Fig. 1). Its deficiency results in both excessive urinary excretion and intralysosomal accumulation of these sulfatides in various tissues (e.g. nervous tissue, gall bladder, kidneys and liver). Especially myelin sheaths of both the central and peripheral nervous system are affected, resulting in progressive demyelination that causes ataxia, initially flaccid and later spastic tetraparesis, mental regression, and other neurological symptoms [3].

Three main clinical types of MLD are distinguished: late-infantile (age of onset before 30 months), juvenile (age of onset between 2.5–16 years) and adult (age of onset after 16 years). Rare congenital and early-infantile types have also been reported [4]. Levels of residual ASA activity correlate with the type and severity of symptoms [5, 6]. Diagnosis of MLD is confirmed by demonstrating deficient ASA activity in leukocytes, increased urinary sulfatide levels, and pathogenic *ARSA* variants. Supportive data include (1) typical brain magnetic resonance imaging (MRI) abnormalities; (2) neurophysiological evidence of a demyelinating sensorimotor polyneuropathy; and (3) neuropsychological evidence of mental regression [7, 8].

At present there is no curative therapy for this devastating disease. However, clinical trials consisting of allogeneic hematopoietic stem cell transplantation (HCT) and gene therapy offer opportunities for presymptomatic or very early symptomatic patients [6, 9, 10]. Nevertheless, treatment effects on peripheral neuropathy are less efficacious compared to effects on brain white matter, especially for HCT [3, 11–13]. The reasons for this are not yet understood. Remarkably, the severity of peripheral neuropathy does often not correlate with the central nervous system (CNS) disease manifestations in untreated patients [14]. Data about the daily impact of peripheral neuropathy in MLD patients are however lacking, since symptomatic patients often show rapid disease progression with dominating CNS symptoms. In this literature review, clinical aspects, pathological findings, distribution of *ARSA* variants, and treatment approaches in MLD are discussed with a particular emphasis on peripheral neuropathy. The full search strategy can be found in Appendix A (Additional file 1).

## The clinical spectrum of metachromatic leukodystrophy

The clinical presentation of MLD is heterogeneous with respect to the age of onset, the speed of progression and the presence of peripheral neuropathy, sometimes even within families [15]. The most prominent peripheral nervous system (PNS) and CNS symptoms of the three MLD types are listed in (Additional file 2: Table S1). In late-infantile MLD patients (48% of MLD patients worldwide and 23% of Dutch MLD patients) [8, 15] the rapidly progressive peripheral neuropathy often precedes the CNS symptoms and is characterized by clumsiness, muscle weakness, sensory deficits and areflexia. Nerve conduction studies demonstrate severe slowing of motor and sensory conduction [16–20]. Nonetheless, as the disease progresses, symptoms of peripheral neuropathy are progressively masked by the development of spastic tetraparesis and other CNS manifestations [21]. Sometimes, the peripheral neuropathy effectively counteracts spasticity. However, in our experience, this is not frequent, especially not in patients with the later onset forms. Other PNS symptoms that we frequently observe in later stages of late-infantile MLD are neurogenic bladder dysfunction, presenting with unexplained signs of discomfort, frequency or retention and sometimes needing intermittent catheterization; neuropathic pain, often responding well on treatment with either amitriptyline or gabapentin; and severe foot deformities.

Contrary to late-infantile MLD, the juvenile type (23% of MLD patients worldwide and 61% of Dutch MLD patients) [8, 15] often begins with cognitive or behavioral disturbances. When comparing with the late-infantile type, signs of peripheral neuropathy, most often areflexia [20], are found less prominent with a lower speed of progression, and more often combined with pyramidal signs and ataxia [22]*.* However, especially the early-juvenile patients may experience severe PNS symptoms as mentioned above, even after treatment with HCT. In the adult variant (22% of MLD patients worldwide and 16% of Dutch MLD patients) [8, 15] psychiatric and behavioral abnormalities are the typical presenting symptoms, with absent peripheral neuropathy or peripheral neuropathy developing in a later stage [23–26]. Areflexia and motor and sensory deficits due to peripheral neuropathy may however be the presenting clinical symptoms in some adult patients [27–33]. In our experience, neuropathic pain, bladder dysfunction and limb deformities due to severe PNS involvement, as seen in the early-onset MLD patients, is rare.

Several studies have addressed the electrophysiological findings of peripheral neuropathy in MLD and their progression over time. A cohort study of 40 MLD patients from India and three case reports found a length dependent neuropathy, in which sensory nerve conduction velocity (NCV) was delayed earlier and more severely than motor NCV [19, 22, 27, 34]. Conversely, the studies of Krishnan et al. [35] and Lütschg [36] found that the motor NCV was more affected than the sensory NCV (39 and four MLD patients respectively)*.* Nonetheless, both motor and sensory NCVs show uniform slowing as is expected for inherited demyelinating polyneuropathies [14, 18–20, 28, 37–39].

## Neuropathology

Histopathologic evaluation of nerve biopsy specimens has been an important diagnostic tool for MLD patients in the past, but can also enhance our understanding of disease pathogenesis nowadays. An overview of the published peripheral nerve abnormalities in different MLD studies is presented in (Additional file 3: Table S2) [5, 31, 42-46, 53, 55-63].

### Accumulation of sulfatides

The accumulation of metachromatic material in peripheral nerves in MLD have first been reported by Jacobi [40]. Metachromatic material consists of Schwann cells and endoneural macrophages that are filled with characteristic lysosomal inclusions of sulfatides, also called inclusion bodies. The sulfatides are normal in structure but cause a lower cerebroside-sulfatide ratio in myelin composition and a disruption in myelin metabolism [41]. Schwann cells and phagocytes die, and demyelination of myelin in the PNS and CNS occurs. Rarely, evidence of actual destruction of the axons can be observed.

Remarkably, no correlation between demyelination and the presence of metachromatic material in peripheral nerves has been found [22, 42–45]. This raises the question whether peripheral neuropathy in MLD is (partially) due to other causes in addition to sulfatide accumulation. On the other hand, sulfatide levels in the cerebrospinal fluid (CSF) and sural nerve do reflect the severity of peripheral neuropathy (measured by nerve conduction studies), while they are not proportional to central white matter injury (assessed by the Gross Motor Function Measure 88–items score, somatosensory evoked potentials, and MR spectroscopy) [42].

### Dynamics in myelin and nerve thickness

Segmental demyelination and reduction in number of myelinated fibers are most severe in late-infantile MLD and in more advanced stages of the disease. Larger myelinated fibers tend to be more affected, resulting into the loss of normal bimodal distribution of myelin sheath thickness. Remyelination may occur and is mostly seen in adult MLD patients. The observed increased g-ratios (ratio between axonal diameter and myelinated fiber diameter) suggest that the thick myelinated fibers are remodeled into thin myelinated fibers [46].

On the other hand, one recent study reported homogenous enlargement of the peripheral nerves on ultrasound in a patient with advanced late-infantile MLD. The echo-intensities of the nerves were normal to reduced, possibly due to the expression of accumulated inclusion bodies [47]. These findings should be taken with caution since they have not been validated in other MLD patients. However, cranial nerve and cauda equina enhancement on MRI might also suggest nerve enlargement secondary to the accumulation of metachromatic material [48–51], although contrast enhancement could also result from a disturbed blood–nerve barrier [52]. Hypertrophic changes and onion bulbs, as are seen in hypertrophic neuropathies and chronic inflammatory demyelinating polyneuropathy, have only rarely been noticed.

### Cell alterations

Inclusion bodies, including zebra, tuffstone, prismatic, lamellar and granular bodies, are the characteristic cell alterations observed in neural and non-neural tissue of MLD patients. They consist of metachromatic material and can already be found in the peripheral nerves of asymptomatic patients, even before birth [53–56]. Numbers of inclusion bodies are higher in patients with late-infantile MLD, due to higher sulfatide levels and lower ASA activity compared to later onset forms. Besides, some studies found that tuffstone bodies are more frequent in late-infantile MLD, while zebra bodies are more frequent in juvenile and adult MLD. However, whether different inclusion body types have different roles in disease pathogenesis is unclear as different types can blend into each other and most likely reflect different orientations and packing of metachromatic material instead of different disease mechanisms [5, 56].

Notably, Cravioto et al. [57] and Argyrakis et al. [53] also described several abnormalities other than inclusion bodies. These are morphological alterations of the endoplasmatic reticulum and mitochondria in Schwann cells, and accumulation of glycogen in mitochondria, Schwann cells and axons. These abnormalities could reflect a metabolic derangement of these cells, causing premature cell death, and may explain the lack of a correlation between demyelination and presence of metachromatic material. Nevertheless, it is too early to draw any firm conclusions based on two individual cases [58].

## Peripheral neuropathy in animal models of metachromatic leukodystrophy

As there is no naturally occurring animal model of MLD, the first *ARSA*-deficient mice were generated by homologous genetic recombination by Hess et al. [64]. Like human patients, the *ARSA*-deficient mice show lipid storage in neuronal and visceral tissues and have impaired hearing and neuromuscular coordination. However, these mice have a normal lifespan without extensive demyelination nor peripheral neuropathy, thus representing a very mild type of MLD [64]. A decade later, Eckhardt and colleagues engineered a new mouse model using transgenic overexpression of ceramide galactosyltransferase selectively in neurons of *ARSA*-deficient mice. This resulted in more pronounced impairment of neuromuscular coordination than in the pure *ARSA*-deficient mice, but still not in frank demyelination nor peripheral neuropathy [65]. As a result, these models do not contribute to our understanding of peripheral neuropathy. However, a novel double-transgenic *mARSA2/2* mouse strain with a demyelinating disease phenotype and reduced NCVs offers new opportunities [66].

What is known about peripheral neuropathy from MLD animal studies comes from an in vivo healthy mouse model constructed by Aguayo and colleagues [67]. They studied myelination of mouse axons in the sciatic nerve by Schwann cells transplanted from human sural nerves of healthy controls and MLD patients. The initial stages of regeneration and myelination were similar in control and MLD nerves, but at two and half months after grafting, numerous metachromatic granules were formed within Schwann cells in the MLD grafts. Since the newly generated nerve fibers within the grafts represented a combination of mouse axons and human Schwann cells, they stated that the grafted MLD sheath cells continued to be ASA deficient during nerve regeneration and that these cells were unable to utilize the enzyme from the mouse.

## Genetics

MLD is caused by variants in the *ARSA* gene on chromosome 22q13.33, which codes for the lysosomal enzyme ASA, or, more rarely, by variants in the *PSAP* gene on chromosome 10q22.1, which codes for the activator protein saposin B. Cesani et al. [15] have described 200 *ARSA* allele variants in 432 MLD patients from 393 families and ten *PSAP* allele variants found in twenty-six patients from eighteen families. They found that approximately 80% of the patients had peripheral neuropathy. Unfortunately, information on electrophysiological findings was only available for 30% of the patients, and the association between genotype and peripheral neuropathy was not studied. However, in earlier reports, an association between the severity of the carried mutation (based on the residual activity of the corresponding enzyme) and peripheral neuropathy was found [68, 69]. In addition, Rauschka et al. [70] observed that peripheral neuropathy is more severe in MLD patients with the homozygous c.1283C > T (p.Pro426Leu) variant (*n* = 22) compared to patients with the heterozygous c.542 T > G (p.Ile181Ser) variant (*n* = 20).

A few studies also suggest an association between genotype and the presence of peripheral neuropathy in adult MLD, although the number of patients included is low. Two variants in the *ARSA* gene are thought to be associated with adult MLD with solely PNS involvement: these are: c.862A > C (p.Thr288Pro, homozygous) [29, 30] and c.1223C3 > T (p.Thr408Ile) [28]. Three other variants in the *ARSA* gene are thought to be associated with adult MLD without PNS involvement: c.661 T > G (p.Phe221Val; homozygous) [25], c.878G > A (p.Arg293Gln) and c.1465 T > G (p.Cys489Gly) [24]. These potential genotype-phenotype associations are interesting as they might help to better predict treatment outcomes.

## Therapeutic approaches

At present, MLD is still a uniformly fatal disease. The genetic and biochemical cause of MLD has facilitated the implementation of a series of clinical studies targeting HCT and gene therapy. However, the striking variation in MLD phenotypes, even within subtypes and families, hampers the possibility to generalize treatment outcomes. Besides that, many clinical studies provide information for only a limited number of patients at various stages of the disease. In general, it appears that asymptomatic patients with juvenile and adult MLD experience a clear symptomatic and survival benefit from allogeneic HCT; however, this benefit is transient and often limited to the CNS symptoms [3, 11, 12, 28, 71–76]. Preliminary studies show that PNS symptoms in patients respond better to gene therapy, most likely due to higher enzyme levels achieved than with HCT and thereby increased penetration into the peripheral nerves [76, 77]. In addition, gene therapy is currently the only treatment that resulted in good functional outcomes for asymptomatic and very early symptomatic patients with late-infantile MLD [78]. Still, no effective treatment has been found for symptomatic MLD patients [3, 79]. For these patients, symptomatic treatments as botulinum toxin or intrathecal administration of baclofen can be helpful to treat spasticity [80]. The following paragraphs provide a summary of the results of preclinical studies and clinical trials targeting HCT, gene therapy, enzyme replacement therapy (ERT), and warfarin administration, with emphasis on the treatment effects on the PNS. (Additional file 4: Table S3) provides a summary of ongoing clinical trials on MLD.

### Allogeneic hematopoietic cell transplantation

Hematopoietic cells from bone marrow, peripheral blood or umbilical cord blood are able to cross the blood-brain and blood-nerve barrier, differentiate into macrophages/microglia, and deliver ASA into the CNS and PNS [76]. Allogeneic HCT has been proven to correct ASA deficiency in MLD patients if stable engraftment following transplantation has been accomplished [79]. Nonetheless, the replacement of ASA deficient host cells by ASA producing donor cells is slow, resulting in a delay estimated at 12–24 months until the disease stabilizes. This makes HCT unsuitable for symptomatic MLD patients or (asymptomatic) patients with the late-infantile MLD. Considering time, unrelated umbilical cord blood is currently preferred over bone marrow and peripheral blood because stored umbilical cord blood can be identified and transplanted faster than other sources [12, 79, 81].

Nevertheless, HCT treatment effects on the PNS in most clinical studies (NCT00383448, NCT00176904, NCT01043640, NCT01626092) are considered disappointing when compared to the CNS, although two case studies describe stabilization or improvement of symptoms in the PNS only [82, 83]. For example, Boucher et al. [11] found that 76% of the patients demonstrated worsening peripheral neuropathy after HCT, compared to 31% of the patients with worsened demyelination in the CNS (*n* = 40, follow-up = 0–30 years). De Hosson et al. [13] found that NCV studies for all patients deteriorated while the white matter lesions on brain MRI were stable for most patients (*n* = 5, follow-up = 18–29 years). Martin et al. [3] evaluated long-term outcomes after unrelated umbilical cord blood transplantation (UCBT) in late-infantile and juvenile MLD patients. They found that the brain lesions improved in 84% of asymptomatic patients, but that the NCV results continued to decrease, resulting in a decline in gross motor function for all except one patient (*n* = 19, follow-up = 2–14 years). Finally, Chen et al. [12] compared asymptomatic juvenile MLD patients who underwent unrelated UCBT. Brain MRI abnormalities were stable, but their peripheral neuropathy progressed. Nonetheless, the speed of progression in UCBT patients was slower when compared with their untreated siblings (*n* = 3, follow-up = 7–17 years).

### Gene therapy

The use of autologous hematopoietic stem cells transduced with a lentiviral vector containing a healthy copy of the *ARSA* gene allows supra-normal production (500–1000%) of ASA by donor cells, due to overexpression of the gene by a stronger promoter. This ex vivo gene therapeutic approach could therefore be faster and more effective in cross correction of ASA deficient graft cells when compared to HCT alone [76, 77]. After favorable treatment effects on both the CNS and PNS in MLD mouse models [84–86], multiple clinical trials on hematopoietic stem cell-directed gene therapy (HSC-GT) for the treatment of MLD have started (NCT02559830, NCT01560182, NCT03392987). The preliminary results and ad-hoc analysis of one of these trials (NCT01560182) have already been published. In this clinical trial, HSC-GT in nine patients with early onset MLD (< 6 years old) in an asymptomatic or early-symptomatic phase, resulted in stable engraftment and correction of ASA deficiency in all hematopoietic cell lines and CSF. At follow-up (18–54 months after HSC-GT), NCV improved in three patients, remained relatively stable in four, and substantially decreased in two, particularly in the first 6–12 months of follow-up. Brain MRI abnormalities were stable or improved in eight patients. Signs of remyelination in the PNS were also found in a few patients, with better remyelination in patients with a higher transduced cell engraftment [78, 87]. Although long-term treatment effects have yet to be determined, the stable or improving NCVs in combination with signs of PNS remyelination indicate that the majority of HSC-GT treated patients do indeed benefit from higher ASA levels, and thereby probably improved enzyme delivery to the PNS when compared to HCT.

Another potential gene therapy approach is to restore the *ARSA* gene in vivo by using an adeno-associated virus (AAV) as vector. This AAV-based gene therapy can be administered directly to the CNS, either via an intraparenchymal or intrathecal route, correcting the *ARSA* gene in local cells and resulting in an even faster ASA expression, secretion and cross-correction in CNS cells, such as astroglial cells and oligodendrocytes for some AAV serotypes serotypes [88–92]. This can be of particular importance since astroglial cells and oligodendrocytes might not take up the non-phosphorylated form of ASA, secreted by bone marrow-derived macrophages/microglia, via the mannose 6-phosphate receptor pathway [93]. Besides, in vivo gene therapy is thought to work at distance, e.g. in the peripheral nerves, by spreading of the AAV vector and/or ASA by either diffusion along the myelinated tracks or by retrograde/anterograde axonal transport [94, 95]. Nevertheless, the potential effects of in vivo gene therapy on the PNS have yet to be demonstrated in MLD. So far, intraparenchymal administration of serotype 5 AAV prevented motor coordination impairment in 18-month-old treated *ARSA* knockout mice, but effects on PNS function could not be judged as also the untreated mice lacked PNS abnormalities [88, 96]. In addition, intraparenchymal administration of serotype 2–5 recombinant AAV did not result in presence of the vector in the sciatic and radial nerves in macaques, whereas a clear diffusion of the vector and a significant increase of ASA activity was observed in the injected brain hemisphere [97]. Finally, a clinical trial on CNS-administered AAV-based gene therapy with serotype rh.10 in human patients with early onset MLD (< 6 years old) (NCT01801709) has been halted due to lack of efficacy [98], and effects on the PNS in these patients have not been reported yet. However, combining CNS-administered and intravenously-administered AAV-based gene therapy might be more promising, as this combination showed synergistic effects on presence of the viral vector, enzyme activity and functional outcomes in both the CNS and PNS in mouse and canine models of Krabbe disease [99, 100].

### Enzyme replacement therapy

ERT is used with variable success in treating some lysosomal disorders, including Gaucher disease, Fabry disease, mucopolysaccharidoses type I, II, and VI, and Pompe disease [101]. However, its applicability to MLD is challenged as ASA has a high molecular weight, and is therefore unable to penetrate the blood-brain and blood-nerve barrier. Nevertheless, Matthes et al. [66] found that intravenous ERT reduced sulfatide storage in the brain and peripheral nerves, and led to increased NCVs in early treated MLD mouse models. Since then, the results of multiple clinical trials on intravenous administration of Metazym (HGT-1111, recombinant human ASA) have been reported (NCT01303146, NCT00681811, NCT00633139, NCT00418561). Unfortunately, none of them show any beneficial treatment effect of ERT on the CNS and PNS in human patients so far [102]. Recently, Simonis et al. [103] were able to increase the catalytic rate constant of intravenous administered ASA by protein engineering, resulting in a threefold more reduction of sulfatide storage in the PNS and CNS in humanized *ARSA* knockout MLD mouse models. This might be promising for all enzyme-based therapies including ERT and gene therapy. In order to avoid the blood-brain barrier, clinical trials consisting of intrathecal administration of recombinant human ASA (HGT-1110) as a for symptomatic late-infantile and juvenile patients (age up to 13 years) have also been started (NCT01510028, NCT01887938); however these results have not been published yet.

### Other therapies

There are several other forms of therapy that have been studied in small clinical trials. One of these is the administration of warfarin. Since vitamin K availability could be a rate-limiting step in the production of sphingolipids and the conversion of cerebrosides into sulfatides [104], it was hypothesized that warfarin, a vitamin K antagonist, could mitigate the MLD phenotype by reducing the amount of sulfatide formation. This hypothesis was supported by the studies of Sundaram and Lev that found that administration of warfarin lowers brain sulfatides in mice [105, 106]*.* Assadi et al. [104] therefore examined the treatment effects of warfarin in four advanced juvenile MLD patients (of whom two patients had a *PSAP* variant; NCT00683189); however, they did not demonstrate any beneficial treatment effects.

The effect of allogeneic mesenchymal stem cell (MSC) infusion was studied in six MLD patients that previously had an allogeneic bone marrow transplantation (no ClinicalTrials.gov identifier). In four of them there was clear evidence of improvement of NCV at follow-up between 1 and 2.5 years, with an increase in NCV between six to 12 m/s. They speculated that this improvement is due to Schwann cell differentiation of MSC in vivo or to passive enzyme transfer into peripheral nerves provided by the MSC. However, MSCs are incapable of differentiating into Schwann cells, and also the transient nature of the improvement in one patient suggests that passive enzyme transfer is more likely [107].

Finally, it was thought that administration of supplemental umbilical cord blood cells would increase the speed at which normal levels of circulating blood cells are re-established after UCBT. This was tested in one clinical trial with ALD-101 in patients with late-infantile and juvenile MLD (NCT00654433), and in one clinical trial with ALD-601 in pregnant women with affected fetuses (NCT01003912). Both studies were terminated early due to disappointing results and non-enrollment, respectively.

## Immunomodulation in metachromatic leukodystrophy

The lack of a correlation between demyelination and the presence of metachromatic material raises the question whether the pathology of peripheral neuropathy in MLD can be partially explained by a neuroinflammatory scenario. Already in 1988, the final degradation product of the third complement component was demonstrated on the surface of myelin sheaths in MLD as in certain known autoimmune neuropathies [108, 109]. One possibility is that complement activation via the alternative pathway amplifies myelin damage in MLD by inducing or enhancing an immune response against myelin [108, 110]. Besides, the accumulation of sulfatides might promote endogenous synthesis and expression of components of the complement pathway in the PNS, which are (partially) regulated by MLD affected Schwann cells and nerve environmental factors [111]. Nevertheless, the latter is speculation, and needs further research on the presence of other complement cleavage products, B lymphocyte activation and antibody production.

**Fig. 2:**
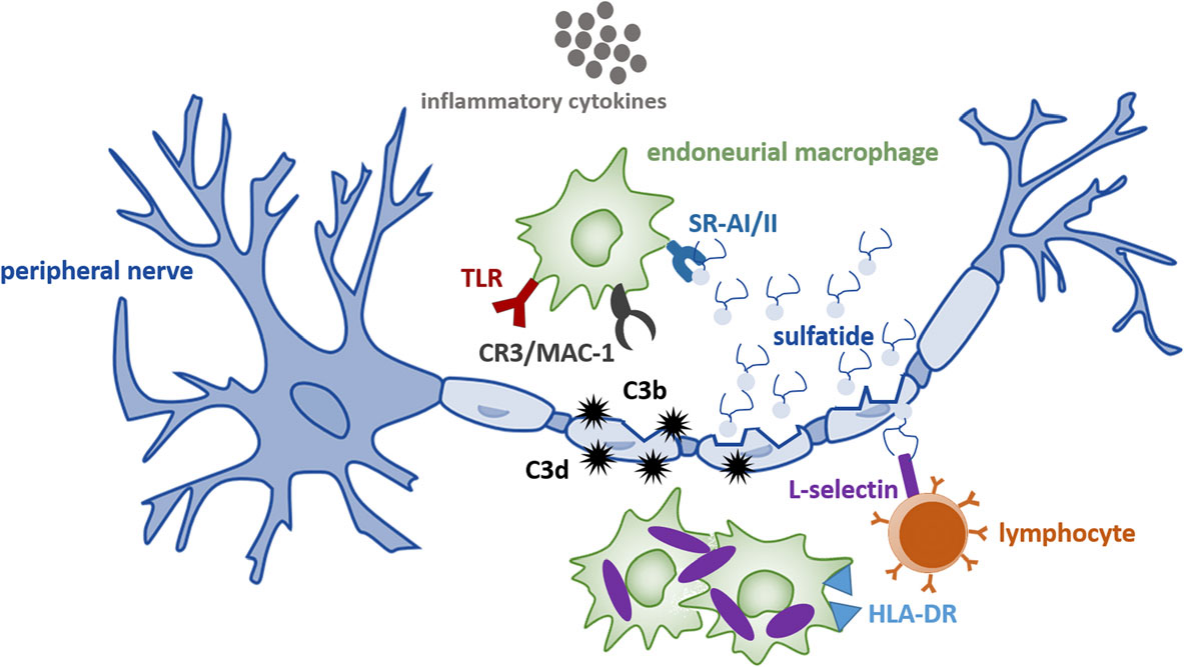
Hypothetical neuroimmune activation model leading to a vicious cycle of demyelination in metachromatic leukodystrophy (MLD). Sulfatide accumulation causes death of Schwann cells and phagocytes, and destruction of myelin in the peripheral nervous system (PNS) [2]. Destruction of myelin activates the third complement component (C3) by the alternative pathway [108, 110], possibly promoted by the disruption of Schwann cells and nerve environment [111] due to sulfatide accumulation. Myelin sheaths are subsequently opsonized by C3b and C3d molecules (hypothetical), which can induce a humoral immune response and act as ‘eat-me’ signals to trigger phagocytosis via the third complement / macrophage-1 receptor (CR3/MAC-1) [133], respectively. In addition, sulfatide accumulation induces the release of pro-inflammatory cytokines and activates endoneural macrophages [106, 107] by acting on the scavenger-receptor-AI/II (SRAI/II) [134]. The released pro-inflammatory cytokines act on the lipophilic receptors (eg. TLR) of endoneural macrophages to assist the phagocytosis of sulfatides and breakdown of myelin sheaths [112]. Macrophage cell death due to the accumulation of sulfatides (shown in purple) also results in presenting sulfatides on their HLD-DR receptors. This assists the activation of lymphocytes that are recruited and activated due to binding of sulfatides and pro-inflammatory cytokines on L-selectin [113]. The activation of lymphocytes in turn leads to cell death and a vicious cycle of demyelination

Additional research findings that suggest a neuroinflammatory component in the pathology of MLD are that sulfatide accumulation and demyelination in the PNS are able to 1) induce the release of inflammatory cytokines; 2) activate endoneural macrophages; and 3) recruit inflammatory myeloid cells and lymphocytes from the periphery [112, 113]. These processes are involved in apoptosis, and could lead to a vicious cycle of demyelination and neuroinflammation as is observed in several other metabolic neurodegenerative diseases like multiple sulfatase deficiency, leukodystrophies like Pelizaeus-Merzbacher disease (PMD), and inherited peripheral neuropathies like Charcot-Marie-Tooth disease type 1B and type 1X [114–116]. A hypothetical neuroimmune activation model leading to a vicious cycle of demyelination in MLD is proposed in Fig. 2.

**Fig. 3:**
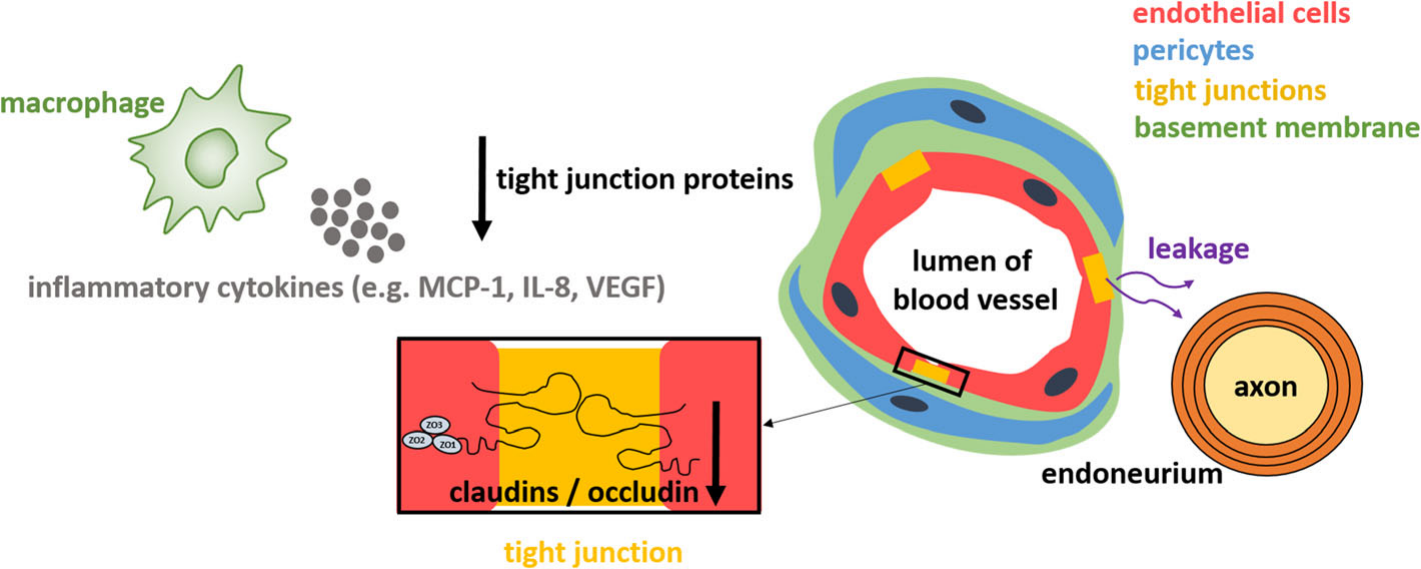
Hypothetical disrupted blood-nerve barrier model leading to leakage of noxious substances from the blood into the endoneurium in metachromatic leukodystrophy (MLD). The release of inflammatory cytokines in MLD, such MCP-1, IL-8 and VEGF, downregulates tight junction proteins, including occludin, claudin-1 and claudin-5 [118]. Consequently, the integrity tight junctions is disrupted, leading to damage of the blood-nerve and leakage of noxious substances from the blood into the endoneurium

Thibert et al. [117] documented significant elevations of MCP-1, IL-1Ra, IL-8, MIP-1b and vascular endothelial growth factor (VEGF) in both CSF and plasma of MLD patients compared to unaffected controls. These inflammatory cytokines are able to disrupt the blood-nerve and blood-brain barrier by downregulating tight junction proteins, causing leakage of noxious substances from the blood into the endoneurium [118] as demonstrated in Fig. 3 for the blood-nerve barrier. The brain might at that point be less susceptible, because the blood-brain barrier contains a protective second basement membrane, the glia limitans perivascularis, and astrocytic endfeet layer, which are both not present in the blood-nerve barrier [119].

Since neuroinflammation could play a role in the pathology of MLD, it is interesting to evaluate the effect of immunomodulatory drugs in MLD patients with a presumptive diagnosis of Guillain-Barré syndrome or chronic inflammatory demyelinating polyneuropathy. A few of them showed short-term functional improvement after treatment with prednisone [120], prednisolone [121], and intravenous immunoglobulins [122, 123]*.* However, contradictory findings have also been described for prednisone [124, 125], and intravenous immunoglobulins [51, 121, 126]. Besides, it cannot be concluded whether immunomodulation altered disease progression or had effects on the function of demyelinated axons [123]. In the latter case, benefits of treatment may be due to stabilization of membrane function, promotion of muscle or neuronal regeneration, or delay in programmed myoblast death, as also seen in muscular dystrophies. A more detailed description of these cases is provided in Appendix B (Additional file 5).

Finally, Thibert et al. [117] found that the elevated plasma levels of MCP-1, IL-1Ra, IL-8 and MIP-1b prior to HCT had decreased by 100 days after HCT (*n* = 1). These data suggest that HCT can alter the state of (neuro-)inflammation in MLD patients, in addition to correcting ASA deficiency, and that this alteration might also have positive effects on demyelination as observed in PMD [115] and MLD mouse models [127]. However, convincing preclinical and clinical evidence for a neuroinflammatory role in the pathology and treatment of peripheral neuropathy in MLD has yet to be shown.

## Conclusions and future directions

Even though multiple treatment strategies have been explored, including ERT, HCT, and HSC-GT, none of them has proven entirely effective in treating MLD patients, with the peripheral demyelination being the most refractory to therapy. Although many clues have emerged from neuropathological, clinical and genetic studies of MLD and other demyelinating storage diseases, the cellular mechanisms of peripheral polyneuropathy in MLD remain elusive. HSC-GT treated patients clearly seem to benefit from the higher achieved ASA levels, and thereby increased penetration into the PNS, when compared to HCT. However, considering MLD not to be caused by only enzyme deficiency and subsequent sulfatide accumulation, but also by an inflammatory component, might provide important insights into the pathophysiology of the disease and the progression of the peripheral neuropathy after treatment. A neuroinflammatory component in the pathology of the disease is an attractive hypothesis with clear therapeutic implications, but convincing preclinical and clinical evidence has yet to be shown. Since *ARSA* knockout mouse models do not show clear demyelination or peripheral neuropathy, the use of double-transgenic mASA2/2 mice [66] is recommended to study inflammation and treatment effects on the PNS.

In order to better understand the clinical impact and possible pathomechanisms of peripheral neuropathy in different stages and forms of MLD, results of repeated PNS measurements in patients, such as nerve conduction velocities, ultrasound and physical exams, could be combined with (historical) nerve pathology findings. Besides, the prevalence and potential role of complement factors and autoantibodies in MLD disease course, e.g. antiganglioside antibodies and anti-myelin-associated glycoprotein antibodies, remains to be explored. Finally, studying whether *ARSA* variants or biomarkers, such as pro-inflammatory cytokines, are correlated to severity of peripheral neuropathy might help to better predict treatment outcomes and select patients for treatment with either HCT (patients with a low chance on severe peripheral neuropathy) or gene therapy (patients with a high chance on severe peripheral neuropathy).

To improve clinical treatment of MLD patients, the management of PNS symptoms should be included as part of the treatment protocol, as these can be severely debilitating even after treatment. Parents of patients should be counseled on the importance of proper footwear and care to prevent deformities, and signs of neuropathic pain and bladder dysfunction that can be treated with amitriptyline or gabapentin and intermittent catheterization, respectively. In addition, a regular (yearly) screening of peripheral neuropathy is advised in symptomatic MLD patients. Simple questionnaires and diagnostic tests such as pin sensibility, strength and tendon reflexes can provide useful information, but will be difficult to perform in severely affected patients. Nerve conduction studies are needed to objectify uniform slowing of both motor and sensory peripheral nerves. As MLD is a disease affecting both the CNS and PNS, it will be challenging to attribute symptoms and signs to either of the two. Following treated patients with stabilization of brain involvement on MRI will be helpful to examine the impact of peripheral neuropathy.

Other important challenges remain also for both clinicians and researchers. Due to the rarity of MLD and the variability in its presentation, many patients are still diagnosed too late to be considered for treatment. International efforts will be necessary to achieve early diagnosis in order to treat these patients and include them in clinical trials at early stages of disease. MLD patients who receive HCT or gene therapy earlier usually have better outcomes than those treated at later stages of the disease [9, 78, 128]. The identification of several elevated sulfatide species, such as C-16-0-OH and C-16-1-OH, as a potential marker of MLD and of disease progression, and the availability of optimized high-throughput assays to measure these in dried blood spots offer possibilities for newborn screening and pre-symptomatic treatment [129]. Currently, a newborn screening pilot study is conducted in Washington State and detected only four false positives out of 70.000 samples [130]. However, additional data on genotype – phenotype relations and biomarkers to predict disease course, and data on (long-term) effects of treating patients earlier in life, especially on preventing peripheral neuropathy in late-infantile patients, are needed to speed up the implementation.

Since there is no universal standard for assessing patients prior to treatment nor for following them after, such data are needed to define the effects and limitations of treatment options. Currently introduced approaches are the MRI MLD score, the MLD gross motor function (for patients from the age of 18 months onwards), and various intelligence tests at a yearly basis for at least 5 years after treatment [9]. However, these assessments predominantly focus on CNS symptoms. For yearly assessing peripheral neuropathy, the Pediatric-modified Total Neuropathy Score [131] in addition to NCV studies may be useful, also for pediatric or incapacitated MLD patients. Nevertheless, this score has not been validated in this specific group of patients yet, and advices on follow-up of peripheral neuropathy in MLD patients are extrapolated primarily from expert opinion.

Finally, especially in cell-based intervention trials like HCT and gene therapy, also clinical trial designs and monitoring protocols should be harmonized to compare study results despite the small numbers and variation in disease phenotypes [132]. Clinical trial protocols, results and neuropathological data should preferably be shared according to the open science approach to advance therapeutic development and increase international collaborations.

## Supplementary information

**Additional file 1:** The full search strategy used to write this systematic review.

**Additional file 2: Table S1.** Clinical spectrum of metachromatic leukodystrophy (MLD). Most prominent symptoms during natural disease course are reported per MLD type. The worldwide contribution of MLD types to all MLD cases is displayed between parentheses in the upper row but varies between different populations.

**Additional file 3: Table S2.** Overview of the noted peripheral nerve abnormalities in metachromatic leukodystrophy (MLD). Findings are presented separately by MLD type and stage of disease whenever possible. The number of studied MLD patients is displayed between parentheses. The number of studied nerves per patient differed between and within reports and is therefore not noted. Type of inclusions are categorized as zebra bodies, tuffstone bodies, lamellar bodies, prismatic bodies, granular bodies by the reviewers based on the descriptions given in the original reports.† the g-ratio is the degree of myelination which is estimated by dividing the axon diameter by the myelinated fiber diameter.*Abbreviations: A-MLD: adult MLD; CNS: central nervous system; J-MLD; juvenile MLD; LI-MLD: late-infantile MLD; PNS: peripheral nervous system.*

**Additional file 4: Table S3.** Ongoing clinical trials on metachromatic leukodystrophy (MLD). A summary of the ongoing clinical trials on treatment for metachromatic leukodystrophy, that are published on https://clinicaltrials.gov/. *Abbreviations: ASA: arylsulfatase A; BMT: bone marrow transplant; cDNA: complementary deoxyribonucleic acid; HSC-GT: hematopoietic stem cell-directed gene therapy; PBSCT: peripheral blood stem cell transplant; HCT: hematopoietic stem cell transplantation; UCB: umbilical cord blood.*

**Additional file 5:** A more detailed description of literature cases treated with immunomodulatory drugs.

## Abbreviations

AAV: Adeno-associated virus
ASA: Arylsulfatase A (enzyme)
CNS: Central nervous system
CSF: Cerebrospinal fluid
E.g.: Exempli gratia
ERT: Enzyme replacement therapy
HCT: Hematopoietic stem cell transplantation
IQ: Intelligence quotient
MLD: Metachromatic leukodystrophy
MRI: Magnetic resonance imaging
MSC: Mesenchymal stem cell
NCV: Nerve conduction velocity
PMD: Pelizaeus-Merzbacher disease
PNS: Peripheral nervous system
UCBT: Umbilical cord blood transplantation
VEGF: Vascular endothelial growth factor
