# Supplementary material for: Peripheral neuropathy in metachromatic leukodystrophy: current status and future perspective
Source: Orphanet J Rare Dis. 2019 Nov 4;14:240. doi: 10.1186/s13023-019-1220-4 (PMC6829806; doi:10.1186/s13023-019-1220-4)
Supplement: Supplementary file 1 — Additional file 1: The full search strategy used to write this systematic review. [file 13023_2019_1220_MOESM1_ESM.docx]

**Appendix A: The full search strategy used to write this systematic review.**

**Pubmed search:** (((((((((((((((((((“Metachromatic Leukodystrophy”[Title/Abstract]) OR “Metachromatic Leucodystrophy”[Title/Abstract]) OR “ARSA Deficiency”[Title/Abstract]) OR “Arylsulfatase A Deficiency”[Title/Abstract]) OR “Cerebroside Sulfatase Deficiency”[Title/Abstract]) OR “Cerebroside 3-Sulfatase Deficiency”[Title/Abstract]) OR “Diffuse Cerebral Sclerosis”[Title/Abstract]) OR “Greenfield Disease”[Title/Abstract]) OR “Greenfield Syndrome”[Title/Abstract]) OR “Metachromatic Leukoencephaly”[Title/Abstract]) OR “Metachromatic Leukoencephalopathy”[Title/Abstract]) OR “Sulfatide Lipidosis”[Title/Abstract]) OR “Sulphatide Lipidosis”[Title/Abstract]) OR “Sulfatidosis”[Title/Abstract]) OR “Cerebroside Sulfate Storage Disease”[Title/Abstract]) OR “Cerebroside Sulphate Storage Disease”[Title/Abstract]) OR “Mckusick 25010”[Title/Abstract]) OR “Leukodystrophy, Metachromatic”[MeSH Terms])) AND (((((((((((((“Peripheral Neuropathy”[Title/Abstract]) OR “Neuropathy”[Title/Abstract]) OR “Peripheral Nervous System Disease”[Title/Abstract]) OR “PNS Disease”[Title/Abstract]) OR “Polyneuropathy”[Title/Abstract]) OR “Neuralgia”[Title/Abstract]) OR “Peripheral Nerve”[Title/Abstract]) OR “Nerve”[Title/Abstract]) OR “Peripheral Nerve Disease”[Title/Abstract]) OR “Peripheral Nerve Disorder”[Title/Abstract]) OR “Peripheral Nervous Disease”[Title/Abstract])) OR “Peripheral Nervous System Diseases”[MeSH Terms])

*Filters: Publication date from 1933/01/01; English*

Search performed on September, 9^th^ 2019, **157 Hits**

**Embase search:** ('metachromatic leukodystrophy':ab,ti OR 'metachromatic leucodystrophy':ab,ti OR 'arsa deficiency':ab,ti OR 'arylsulfatase a deficiency':ab,ti OR 'cerebroside sulfatase deficiency':ab,ti OR 'cerebroside 3-sulfatase deficiency':ab,ti OR 'diffuse cerebral sclerosis':ab,ti OR 'greenfield disease':ab,ti OR 'greenfield syndrome':ab,ti OR 'metachromatic leukoencephaly':ab,ti OR 'metachromatic leukoencephalopathy':ab,ti OR 'sulfatide lipidosis':ab,ti OR 'sulphatide lipidosis':ab,ti OR 'sulfatidosis':ab,ti OR 'cerebroside sulfate storage disease':ab,ti OR 'cerebroside sulphate storage disease':ab,ti OR 'mckusick 25010':ab,ti OR 'metachromatic leukodystrophy'/de) AND ('peripheral neuropathy':ab,ti OR 'neuropathy':ab,ti OR 'peripheral nervous system disease':ab,ti OR 'pns disease':ab,ti OR 'polyneuropathy':ab,ti OR 'neuralgia':ab,ti OR 'peripheral nerve':ab,ti OR 'nerve':ab,ti OR 'peripheral nerve disease':ab,ti OR 'peripheral nerve disorder':ab,ti OR 'peripheral nervous disease':ab,ti OR 'peripheral nervous system diseases'/de) AND [english]/lim AND [1-1-1933]/sd NOT [1-1-3001]/sd NOT ‘suppl’ NOT ‘conference abstract’

*Filters: Publication date from 1933/01/01; English;*  Not: supplementary material/conference abstract

Search performed on September, 9^th^ 2019, **183 Hits**

**Cochrane search:** Metachromatic Leukodystrophy AND Peripheral Neuropathy

Search performed on September, 9^th^ 2019, **0 Hits**

**Total** **from search =** 340 papers

**Total after removing 140 duplicates =** 200 papers

**Screening on title/abstract**

- **Inclusion =** 71 papers
- **Exclusion =** 129 papers
  - Letters, commentaries, supplementary material, conference abstracts, poster presentations, editorials (n = 6)
  - No abstract available (not clear on title only if should be included) (n = 2)
  - Studies not on metachromatic leukodystrophy (i.e. Hurler syndrome) (n = 51)
  - Reviews/articles on treatment/management of metachromatic leukodystrophy, not focusing on peripheral neuropathy (i.e. opticus neuropathy, central nervous system) (n = 70)

**Screening on full text**

- **Inclusion =** 63 papers
- **Exclusion =** 8 papers
  - No full text available (n = 7)
  - No additional info on MLD (n = 1)

**Cross-referencing**

- **Inclusion =** 11 papers

**Total papers in systematic review =** 134 papers

- Total included from search: 63 (search string) + 11 (cross-referencing) = 74 papers
- Total included for background information = 60 papers
