# Supplementary material for: Peripheral neuropathy in metachromatic leukodystrophy: current status and future perspective
Source: Orphanet J Rare Dis. 2019 Nov 4;14:240. doi: 10.1186/s13023-019-1220-4 (PMC6829806; doi:10.1186/s13023-019-1220-4)
Supplement: Supplementary file 2 — Additional file 2: Table S1. Clinical spectrum of metachromatic leukodystrophy (MLD). Most prominent symptoms during natural disease course are reported per MLD type. The worldwide contribution of MLD types to all MLD cases is displayed between parentheses in the upper row but varies between different populations. [file 13023_2019_1220_MOESM2_ESM.docx]

**Table S1: Clinical spectrum of metachromatic leukodystrophy (MLD).**

|  | **Late-infantile type (48%)** | **Juvenile type (23%)** | **Adult type (22%)** |
| --- | --- | --- | --- |
| Age at onset of symptoms | 6 months – 2.5 years | 2.5 – 16 years | After 16 years |
| Initial symptoms | Peripheral neuropathy with clumsiness, muscle weakness, sensory deficits and areflexia, motor regression, ataxia | Deterioration of school performance, behavioral disturbances, less prominent peripheral neuropathy and often combined with a mild pyramidal syndrome and ataxia | Prominent intellectual and behavioral changes often accompanied by other psychiatric symptoms, such as delusions; presentation with peripheral neuropathy also possible |
| Subsequent symptoms | Mental regression, spastic tetraparesis, visual and auditory impairment, bulbar palsy, seizures (25 – 50% of the cases, mostly grand mal seizures) | Mental regression, spastic tetraparesis, incontinence, optic atrophy, bulbar palsy, seizures (50 – 60% of the cases, mostly complex partial seizures) | Spastic tetraparesis, incontinence, choreiform movements, bulbar palsy, dementia, seizures (2 – 10% of the cases) |
| End stage | Severely disabled state with loss of all motor functions and speech, and death within 1 – 7 years after onset | Severely disabled state with loss of all motor functions and speech, and death within 3 – 15 years after onset | Severely disabled state with loss of all motor functions and speech, and death within 5 – 35 years after onset |

Per MLD type, the most prominent symptoms during natural disease course are reported. The worldwide contribution of MLD types to all MLD cases is displayed between parentheses in the upper row but varies between different populations.
