# Supplementary material for: Peripheral neuropathy in metachromatic leukodystrophy: current status and future perspective
Source: Orphanet J Rare Dis. 2019 Nov 4;14:240. doi: 10.1186/s13023-019-1220-4 (PMC6829806; doi:10.1186/s13023-019-1220-4)
Supplement: Supplementary file 3 — Additional file 3: Table S2. Overview of the noted peripheral nerve abnormalities in metachromatic leukodystrophy (MLD). Findings are presented separately by MLD type and stage of disease whenever possible. The number of studied MLD patients is displayed between parentheses. The number of studied nerves per patient differed between and within reports and is therefore not noted. Type of inclusions are categorized as zebra bodies, tuffstone bodies, lamellar bodies, prismatic bodies, granular bodies by the reviewers based on the descriptions given in the original reports.† the g-ratio is the degree of myelination which is estimated by dividing the axon diameter by the myelinated fiber diameter.Abbreviations: A-MLD: adult MLD; CNS: central nervous system; J-MLD; juvenile MLD; LI-MLD: late-infantile MLD; PNS: peripheral nervous system. [file 13023_2019_1220_MOESM3_ESM.docx]

**Table S2: Overview of the noted peripheral nerve abnormalities in metachromatic leukodystrophy (MLD)**

| **Study** | **MLD type** | **Stage of disease** | **Type of inclusions** | **Other pathological findings** |
| --- | --- | --- | --- | --- |
| Argyrakis et al. (1977) [53] | A-MLD (n = 1) | Asymptomatic (2 – 3 decades before clinical onset of the disease could be expected based on family history) | Zebra bodies  Tuffstone bodies  Lamellar bodies  Granular bodies | Early segmental demyelination  Several myelin ovoids  Rare axonal destruction  Dilated endoplasmic reticulum, altered mitochondria and glycogen accumulation |
| Bardosi et al. (1987) [46] | LI-MLD (n = 4)  J-MLD (n = 2)  A-MLD (n = 3) | Not specified | Zebra bodies  Tuffstone bodies  Prismatic bodies | Reduced myelin sheath thickness, particularly for the thick myelinated fibers Increased g-ratios†, most severe in J-MLD and A-MLD  Loss of normal bimodal distribution of myelin sheath thickness only in LI-MLD |
| Bindu et al. (2005) [22] | LI-MLD  J-MLD  (total n = 40) | Not specified | Lamellar bodies  Prismatic bodies  Granular bodies | Reduced number of myelinated fibers  Diffuse, uniform hypomyelination with abnormal myelin configuration and remodeling  No correlation between demyelination and age ate onset or stage of disease  No onion bulbs  Loss of both large and small diameter axons |
| Cravioto et al. (1966) [57] | J-MLD (n = 1) | Advanced (lost ability to walk unaided) | Tuffstone bodies  Lamellar bodies  Granular bodies | Complete degeneration of some nerve fibers, including the axis-cylinders  Myelin ovoids  Abnormal mitochondria |
| Dayan (1967) [43] | J-MLD (n = 1) | Postmortem | Not described | Segmental demyelination and remyelination  Reduced number of myelinated fibers  Many myelin ovoids  No correlation between demyelination and the presence of metachromatic material  No axonal destruction |
| Dali et al. (2015) [42] | LI-MLD (n = 12) | 10 patients advanced MLD (lost ability to walk unaided) | Not described | Reduced number of myelinated fibers  Reduced myelin sheath thickness, particularly for the thick myelinated fibers  Increased g-ratios†  Loss of normal bimodal distribution of myelin sheath thickness  No axonal destruction |
| De Webster (1962) [44] | LI-MLD (n = 1) | Advanced (spasticity and epilepsy) | Lamellar bodies  Tuffstone bodies | Segmental demyelination  Myelin ovoids  No correlation between demyelination and the presence of metachromatic material |
| Di Trapani et al. (1979) [59] | J-MLD (n = 1) | Advanced (spasticity, unable to maintain position without double support) | Zebra bodies  Tuffstone bodies  Lamellar bodies  Granular bodies | Segmental demyelination without signs of remyelination  Reduced number of myelinated fibers  Onion bulbs and signs of Wallerian degeneration (not prominent) |
| Fenzi et al. (1983) [60] | LI-MLD (n = 1) | Advanced (lost ability to walk unaided) | Zebra bodies  Tuffstone bodies  Lamellar bodies | Segmental demyelination and remyelination  Reduced number of myelinated fibers |
| Fressinaud et al. (1992) [31] | A-MLD (n = 1) | Intermediate (4 years after clinical onset without CNS involvement) | Lamellar bodies  Prismatic bodies | Segmental demyelination and remyelination  1% axonal destruction |
| Guzzetta et al. (1995) [61] | LI-MLD (n = 2)  J-MLD (n = 3) | Not specified | Tuffstone bodies  Prismatic bodies | Segmental demyelination and remyelination  Relative loss of larger myelinated fibers but normal total number of myelinated fibers  Several onion bulbs  Loss of normal bimodal distribution of myelin sheath thickness only in J-MLD |
| Hageman et al. (1995) [62] | A-MLD (n = 7) | Not specified | Zebra bodies  Tuffstone bodies | Segmental demyelination and remyelination, less active compared to LI-MLD and J-MLD  Reduced myelin sheath thickness, particularly for the thick myelinated fibers  Reduced number of myelinated fibers  Slight onion bulb formation |
| Joosten et al. (1975) [45] | A-MLD (n = 1) | Postmortem | Zebra bodies  Lamellar bodies | Slight signs of demyelination  No correlation between demyelination and the presence of metachromatic material |
| Luijten et al. (1978) [5] | LI-MLD (n = 1)  J-MLD (n = 1)  A-MLD (n = 1) | Advanced in the LI-MLD and J-MLD patients (tetraparesis); and intermediate in the A-MLD patient (within 1 year after clinical onset) | Zebra bodies (not in LI-MLD)  Tuffstone bodies  Lamellar bodies  Prismatic bodies (only in J-MLD)  Granular bodies | Segmental demyelination, most severe in LI-MLD and A-MLD  Reduced number of myelinated fibers, most severe in LI-MLD  Axonal destruction only in A-MLD |
| Martin et al. (1982) [55] | LI-MLD (n = 5)  J-MLD (n = 2)  A-MLD (n = 2) | Advanced (extensive CNS and PNS symptoms); and postmortem | Zebra bodies  Tuffstone bodies  Lamellar bodies  Prismatic bodies | Segmental demyelination and reduced myelin sheath thickness, particularly for the largest axons, and most severe in LI-MLD and later disease stages  Loss of normal bimodal distribution of myelin sheath thickness only in LI-MLD  No axonal destruction |
| Meier et al. (1976) [56] | LI-MLD (n = 1) | Asymptomatic (fetus) | Tuffstone bodies  Lamellar bodies | Similar onset and degree of myelination as in a healthy fetus of the same age |
| Percy et al. (1977) [63] | LI-MLD (n = 2)  A-MLD (n = 3) | Intermediate in one A-MLD patient (mental decline only); and advanced in the other patients (extensive CNS and PNS symptoms) | Zebra bodies  Lamellar bodies | Segmental demyelination, most severe in LI-MLD, with more remyelination in A-MLD  Reduced number of myelinated fibers and smaller myelinated fibers, most severe in A-MLD |
| Thomas et al. (1977) [58] | LI-MLD (n = 1)  J-MLD (n = 3) | Advanced (extensive CNS and PNS symptoms) | Zebra bodies  Tuffstone bodies  Lamellar bodies  Prismatic bodies  Granular bodies | Hypertrophic changes and onion bulbs, most prevalent in the later onset J-MLD  Axonal destruction only in the later onset J-MLD |
| Findings are presented separately by MLD type and stage of disease whenever possible. The number of studied MLD patients is displayed between parentheses. The number of studied nerves per patient differed between and within reports and is therefore not noted. Type of inclusions are categorized as zebra bodies, tuffstone bodies, lamellar bodies, prismatic bodies, granular bodies by the reviewers based on the descriptions given in the original reports.  † The g-ratio is the degree of myelination which is estimated by dividing the axon diameter by the myelinated fiber diameter  *Abbreviations: A-MLD: adult MLD; CNS: central nervous system; J-MLD; juvenile MLD; LI-MLD: late-infantile MLD; PNS: peripheral nervous system* | | | | |
