# Supplementary material for: Peripheral neuropathy in metachromatic leukodystrophy: current status and future perspective
Source: Orphanet J Rare Dis. 2019 Nov 4;14:240. doi: 10.1186/s13023-019-1220-4 (PMC6829806; doi:10.1186/s13023-019-1220-4)
Supplement: Supplementary file 4 — Additional file 4: Table S3. Ongoing clinical trials on metachromatic leukodystrophy (MLD). A summary of the ongoing clinical trials on treatment for metachromatic leukodystrophy, that are published on https://clinicaltrials.gov/. Abbreviations: ASA: arylsulfatase A; BMT: bone marrow transplant; cDNA: complementary deoxyribonucleic acid; HSC-GT: hematopoietic stem cell-directed gene therapy; PBSCT: peripheral blood stem cell transplant; HCT: hematopoietic stem cell transplantation; UCB: umbilical cord blood. [file 13023_2019_1220_MOESM4_ESM.docx]

**Table S3: Ongoing clinical trials on metachromatic leukodystrophy (MLD)**

| **Treatment** | **Mode of action** | **Form** | **Phase** | **ClinicalTrials.gov identifier** |
| --- | --- | --- | --- | --- |
| SHP611 (formerly HGT-1110) | Intrathecal recombinant human ASA substitution (biological) | Late-infantile | 2 | NCT03771898 |
| TYF-ARSA | Intracerebral administration of a lentiviral vector carrying a functional *ARSA* gene (gene therapy) | All forms | 1/2 | NCT03725670 |
| AAVrh.10cuARSA | Intracerebral administration of an adeno-associated virus serotype to transfer *ARSA* cDNA into the brain (gene therapy) | Late-infantile  Early juvenile (<5 years) | 1/2 | NCT01801709 |
| HGT-1110 | Intrathecal recombinant human ASA substitution (biological) | Late-infantile | 1/2 | NCT01887938 |
| CD34+ stem cells transduced with *ARSA* encoding lentiviral vector | *ARSA* gene transfer into hematopoietic stem/progenitor cells with a lentiviral vector (Autologous HSC-GT) | Late-infantile  Early juvenile | 1/2 | NCT02559830 |
| CD34+ stem cells transduced with *ARSA* encoding lentiviral vector | *ARSA* gene transfer into hematopoietic stem/progenitor cells with a lentiviral vector (Autologous HSC-GT) | Late-infantile  Early juvenile | 1/2 | NCT01560182 |
| GSK2696274 | Autologous cluster of CD34+ stem cells transduced with a lentiviral vector containing human *ARSA* cDNA (Autologous HSC-GT) | Late-infantile  Early juvenile (<6 years) | 3 | NCT03392987 |
| BPX-501 | Infusion of BPX-501 gene modified T cells (Rivogenlecleucel: biological) and Rimiducid (drug) after allogenic SCT | Not specified | 1/2 | NCT03639844 |
| Busulfan Fludarabine | Hematopoietic SCT using different busulfan- and fludarabine-based conditioning regimens | All forms | 2 | NCT02171104 |
| Human Placental Derived Stem Cell | Human placental-derived stem-cells combined with unrelated and related cord blood SCT | All forms | 1 | NCT01586455 |
| Enriched Hematopoietic Stem Cell Infusion | A second dose of facilitating cell-enhanced hematopoietic stem cell product after hematopoietic SCT (biological) | All forms | 1/2 | NCT01372228 |
| DUOC-01 | Unrelated UCB transplantation with administration of intrathecal UCB derived oligodendrocyte-like cells (DUOC-01) | All forms | 1 | NCT02254863 |
| MGTA-456 / HSC835 | Unrelated UCB transplantation with administration of an expanded UCB product (MGTA-456) | Late-infantile  Juvenile | 2 | NCT03406962 |
| Hydroxyurea, Alemtuzumab, Fludarabine, Melphalan, Thiotepa | Reduced-intensity condition regimens with UCB transplant, double cord UCB transplantation, matched unrelated donor BMT or PBSCT | All forms | 2 | NCT01962415 |
| A summary of the ongoing clinical trials on treatment for metachromatic leukodystrophy, that are published on <https://clinicaltrials.gov/>.  *Abbreviations: ASA: arylsulfatase A; BMT: bone marrow transplant; cDNA: complementary deoxyribonucleic acid; HSC-GT: hematopoietic stem cell-directed gene therapy; PBSCT: peripheral blood stem cell transplant; HCT: hematopoietic stem cell transplantation; UCB: umbilical cord blood* | | | | |
