# Supplementary material for: Peripheral neuropathy in metachromatic leukodystrophy: current status and future perspective
Source: Orphanet J Rare Dis. 2019 Nov 4;14:240. doi: 10.1186/s13023-019-1220-4 (PMC6829806; doi:10.1186/s13023-019-1220-4)
Supplement: Supplementary file 5 — Additional file 5: A more detailed description of literature cases treated with immunomodulatory drugs. [file 13023_2019_1220_MOESM5_ESM.docx]

**Appendix B: A more detailed description of literature cases treated with immunomodulatory drugs.**

Aziz and Pearce [120] described a patient with late-infantile MLD and the inability to support herself unaided, who was given prednisone 5mg twice a day. She showed a definite but slight improvement in the following three weeks and at the time of her discharge from hospital she could even walk. However, while at home the prednisone was increased to 30mg daily, her neurological condition soon deteriorated again and she died of bronchopneumonia one year after disease onset.

On the other hand, Yudell et al. [124] described a patient with juvenile MLD who received prednisone orally for two months. Her symptoms deteriorated without evidence for short-term improvement of symptoms. In addition, Stoeck et al. [125] described a 48-year-old MLD patient who presented with personality changes, progressive memory loss and a mild demyelinating sensorimotor polyneuropathy. He was treated with 1g intravenous methylprednisolone per day for three days, followed by 100mg per day oral prednisolone, tapered down to 20mg over four weeks, and showed also no treatment response.

Haberlandt et al. [121] described two patients with juvenile MLD who both presented with a demyelinating polyneuropathy in the absence of brain abnormalities and who were subsequently treated with immunomodulatory drugs. Patient one was a 2-year old boy who was unable to stand. He was treated with prednisolone with short-term improvement of symptoms but was then lost to follow-up for six years. When he returned to the hospital, he had severe dystrophy, tetraparesis, swallowing problems and bilateral optic atrophy. He died at the age of ten years due to an aspiration pneumonia. The second patient was an 18-month old girl with weakness, muscle atrophy and reduced deep tendon reflexes in the lower extremities. Muscle and nerve biopsy showed neuropathic changes and signs of subacute de- and remyelination without inflammatory infiltrates. She was given two courses of intravenous immunoglobulin without clinical improvement. She also died due to pneumonia at the age of almost four years.

Intravenous immunoglobulins were also given to a patient with late-infantile MLD in whom demyelinating peripheral neuropathy was the presenting feature [126]. Physical examination at that time revealed normal mental status, lower facial weakness, dysphagia and frequent drooling, diffuse extremity weakness (distal greater than proximal), and absent reflexes. Her clinical course however continued to worsen. After seven months she had lost the ability to sit and had developed impaired feeding and loss of meaningful speech.

Dubey et al. [122] describe a 1.5-year old girl with late-infantile MLD who was treated with intravenous immunoglobulins when she presented with an acute-onset flaccid tetraparesis, ptosis, and respiratory failure. A sural nerve biopsy demonstrated demyelinating polyneuropathy with metachromatic granules within Schwann cells. Ptosis and respiratory failure responded completely to treatment, whereas tetraparesis showed minimal improvement. No further follow-up data is reported. Although it is possible that she presented with MLD that responded well to immunoglobulins, the acute presentation combined with respiratory failure makes an episode of Guillain-Barré syndrome in a patient with underlying MLD more likely.

Roi et al. [51] reported a 2-year old late-infantile MLD patient who presented with a demyelinating polyneuropathy and an MRI scan suggestive of delayed myelination or hypo-myelination without other specific features. She was treated with two courses of immunoglobulin and steroids for a presumptive diagnosis of CIDP, but no improvement was observed.

Finally, Nevo et al. [123] described a 4-year old patient with juvenile MLD in whom immunomodulation (consisting of treatment with human immunoglobulins for two months, followed by treatment with cyclosporine A and azathioprine for 19 months) given early in disease course resulted in temporary functional improvement. However, they could not conclude whether the immunomodulation altered the disease progression or had direct effects on the function of the demyelinated axons. In the latter case, benefits of treatment may be due to stabilization of membrane function, promotion of muscle or neuronal regeneration, or delay in programmed myoblast death, as is seen in muscular dystrophies.

**References**

51. Roi D, Mankad K, Kaliakatsos M, Cleary M, Manzur A, D'Arco F. Thickening of the optic nerves in metachromatic leucodystrophy: A new MRI finding. Neuroradiol J. 2016;29(2):134-6.

120. Aziz H, Pearce J. Peripheral neuropathy in metachromatic leukodystrophy. British Medical Journal. 1968;4:300.

121. Haberlandt E, Scholl-Bürgi S, Neuberger J, Felber S, Gotwald T, Sauter R, et al. Peripheral neuropathy as the sole initial finding in three children with infantile metachromatic leukodystrophy. Eur J Paediatr Neurol. 2009;13(3):257-60.

122. Dubey R, Chakrabarty B, Gulati S, Sharma MC, Deopujari S, Baheti N, et al. Leukodystrophy presenting as acute-onset polyradiculoneuropathy. Pediatr Neurol. 2014;50(6):616-8.

123. Nevo Y, Pestronk A, Lopate G, Carroll SL. Neuropathy of metachromatic leukodystrophy: improvement with immunomodulation. Pediatr Neurol. 1996;15(3):237-9.

124. Yudell A, Gomez MR, Lambert EH, Dockerty MB. The neuropathy of sulfatide lipidosis (metachromatic leukodystrophy). Neurology. 1967;17(2):103-11 passim.

125. Stoeck K, Psychogios MN, Ohlenbusch A, Steinfeld R, Schmidt J. Late-Onset Metachromatic Leukodystrophy with Early Onset Dementia Associated with a Novel Missense Mutation in the Arylsulfatase A Gene. J Alzheimers Dis. 2016;51(3):683-7.

126. Gonorazky HD, Amburgey K, Yoon G, Vajsar J, Widjaja E, Dowling JJ. Subacute demyelinating peripheral neuropathy as a novel presentation of late infantile metachromatic leukodystrophy. Muscle Nerve. 2017;56(5):E41-E4.
